# Supplementary material for: Electrical appliances moderate households’ water demand response to heat
Source: Nat Commun. 2018 Dec 20;9:5408. doi: 10.1038/s41467-018-07833-3 (PMC6302094; doi:10.1038/s41467-018-07833-3)
Supplement: Supplementary file 1 — Supplementary Information [file 41467_2018_7833_MOESM1_ESM.pdf]

File name: Supplementary Information

Description: Supplementary Figures and Supplementary Tables

File name: Peer Review File

Description: Supplementary Information for Electrical Appliances Moderate Households' Water Demand Response to Heat, by Alberto Salvo

Supplementary Fig. 1. Income and apartment type

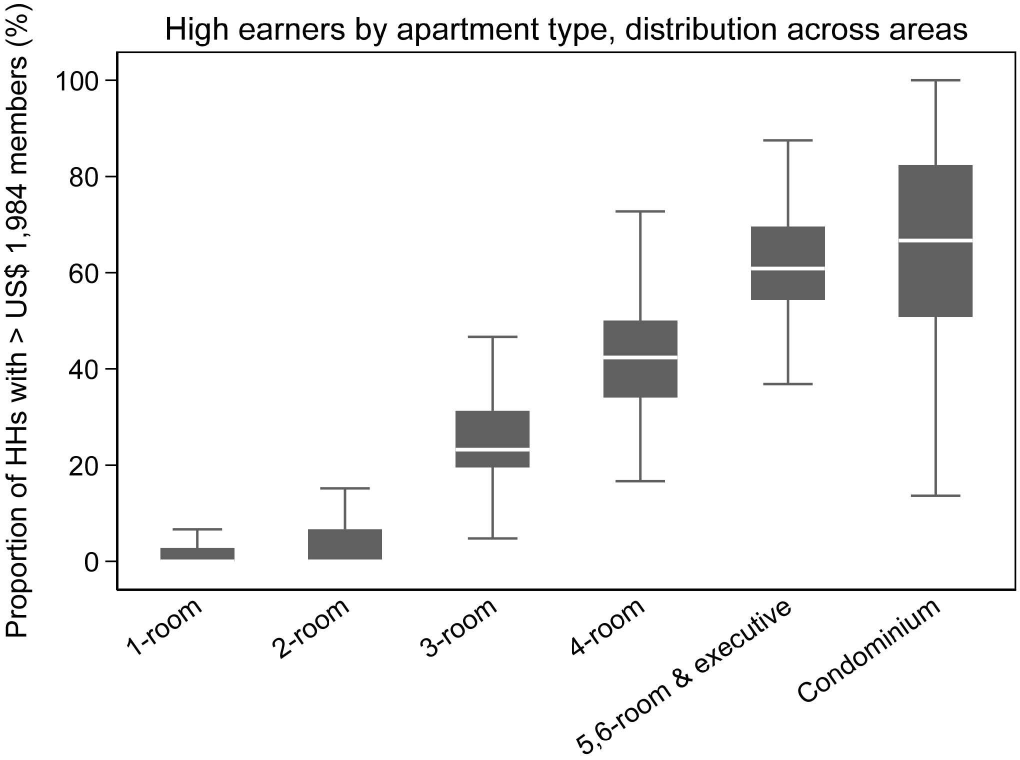

Proportion of households where at least one member states earning over US$ 1,984 month^{-1}, by apartment type. An observation is a geographic area of Singapore, specifically, the 63 residential (two-digit) postal codes covered in the HITS. The box plot shows the median and interquartile range (25th percentile to 75th percentile in the thick bands) in the distribution over geographic areas, by apartment type, for the proportion of households where one or more members stated monthly income of at least SG$ 2,500 (or US$ 1,984). Source: Land Transport Authority's 2008 Household Interview Travel Survey (HITS).

Supplementary Fig. 2. Ground-level wind patterns in Singapore

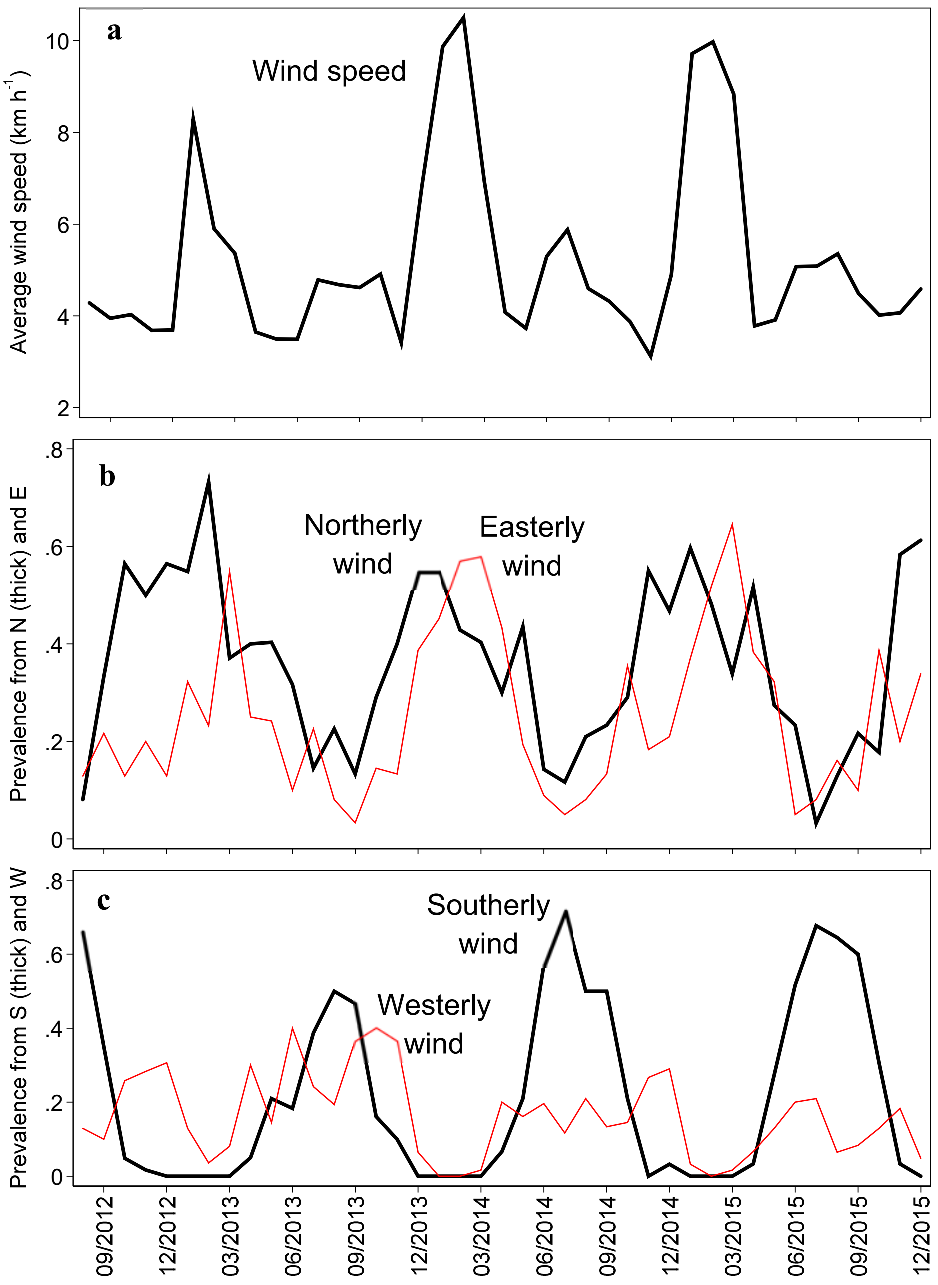

a., Average wind speed (km h^{-1}), **b.**, Prevalence of wind from north (thick black line) and east (thin red line), **c.**, Prevalence of wind from south (thick black line) and west (thin red line). The sample period is August 1, 2012 to December 31, 2015. For each day in the sample, "wind direction from north" is assigned: (i) a value of 1 when recorded wind direction (at 8 am, local time) lies between 330 degrees (from north, clockwise, direction from which the wind blows) and 30 degrees; (ii) a value of 0.5 when wind direction lies between 300 and 330 degrees or between 30 and 60 degrees; and (iii) a value of 0 otherwise. The other three wind direction controls are defined analogously. For better visualization, the plots show means over days within year by month. Source: NOAA Integrated Global Radiosonde Archive (IGRA Version 2).

Supplementary Fig. 3. Distribution of the number of usage periods by household in the sample.

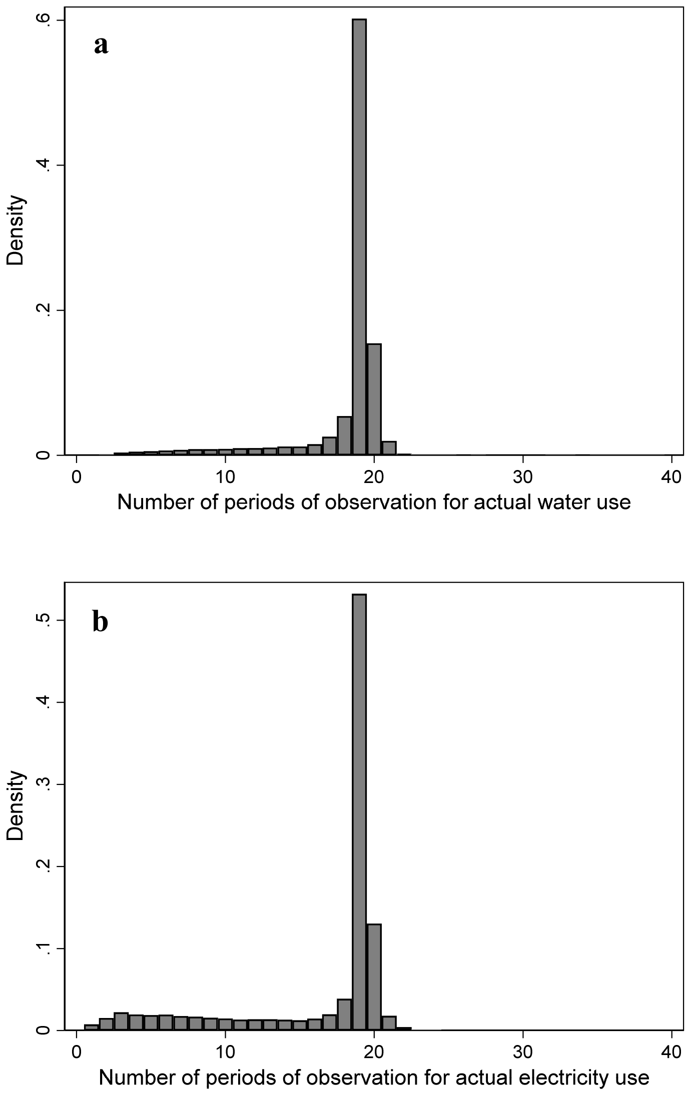

Number of usage periods by household for **a.,** water, and **b.,** electricity. An observation is a household. Source: SP Services microdata.

Supplementary Fig. 4. Allowing for a nonlinear response in the household demand model

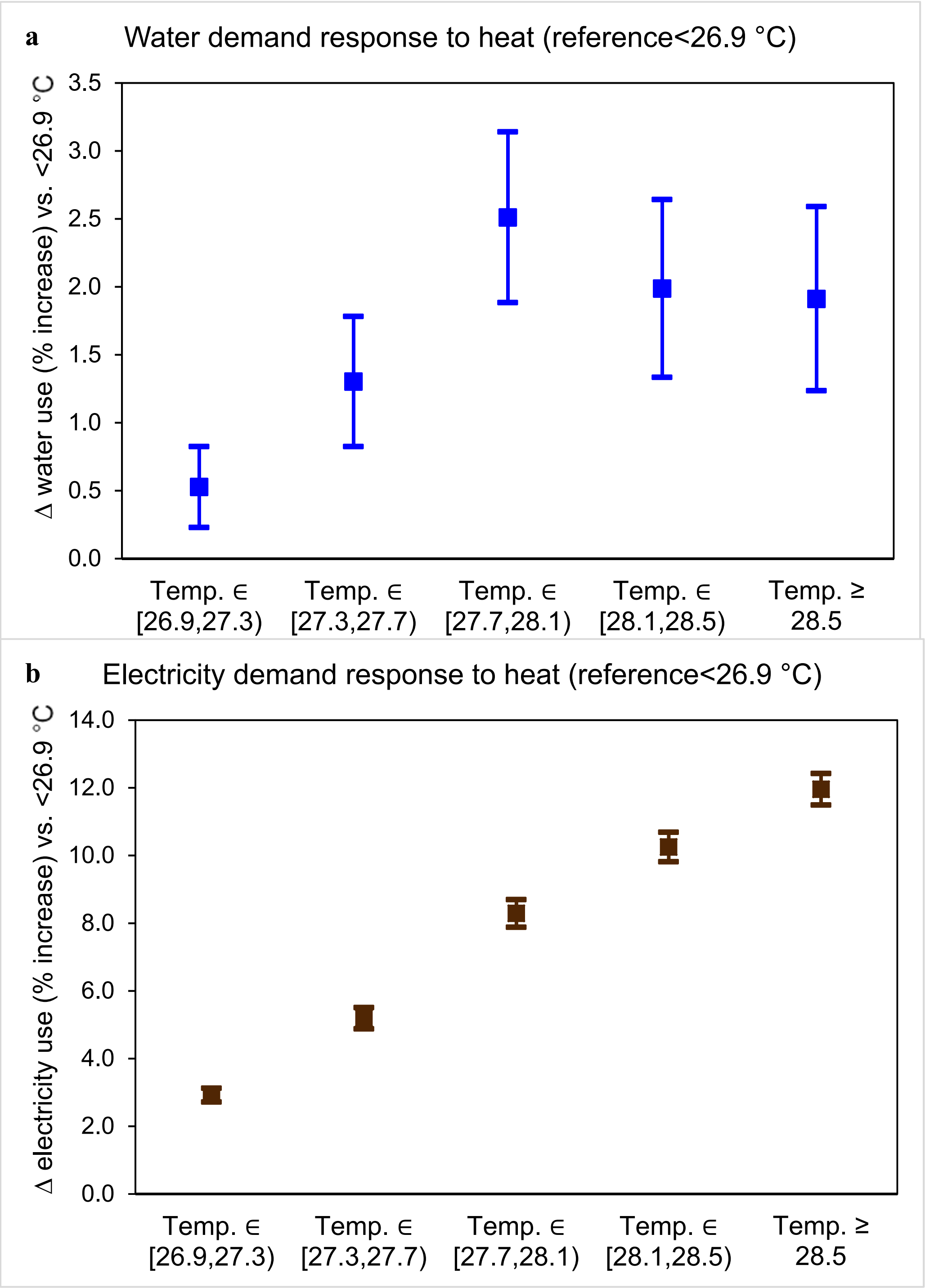

95% confidence intervals for the coefficients on average temperature bins, converted to percent increase in use, obtained from two separate OLS regressions: **a.,** log water use (m^3 $month^{-1}$), and **b.,** log electricity use (kWh $month^{-1}$). The regression models and samples follow that in specification 4 of Table 1 in the main text, with one exception: the variable of interest, average temperature over the usage period, enters flexibly via bins (e.g., between 26.9 °C and 27.3 °C),

rather than linearly. The reference category is average temperature < 26.9 °C. The empirical support for average temperature is 26.1 to 29.1 °C. Regressions include controls for household, time (month, year, day-of-the-week, public holidays, school holidays), weather (relative humidity, dew point depression, wind speed, precipitation) and PM2.5.

Supplementary Fig. 5. Further robustness tests

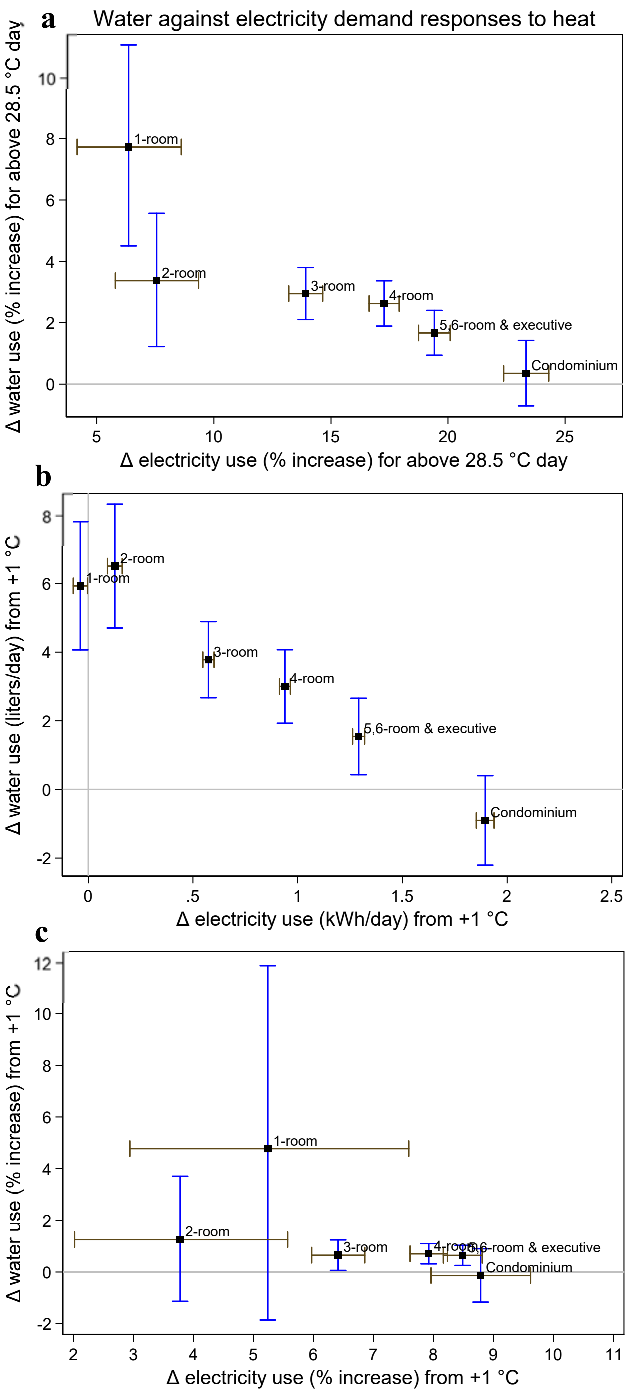

Robustness to: **a.,** Temperature variable, based on the proportion of days in the usage period with daily mean temperature above 28.5 °C, as in Supplementary Table 4, rather than the average temperature in the usage period. **b.,** Dependent variables in levels, namely water use (converted to L day^{-1}) and electricity use (kWh day^{-1}), rather than the natural logarithm of resource use. **c.,** Estimation by apartment type subsample: 2 resources × 6 apartment types = 12 separate OLS

regressions of log water use or log electricity use. 95% confidence interval for the percent increase from raising the temperature variable by 1 unit (0 to 100% in **a**, +1 °C in **b** and **c**). Unless noted, specifications follow that in Fig. 5a in the main text.

Supplementary Fig. 6. Importance of environmental controls.

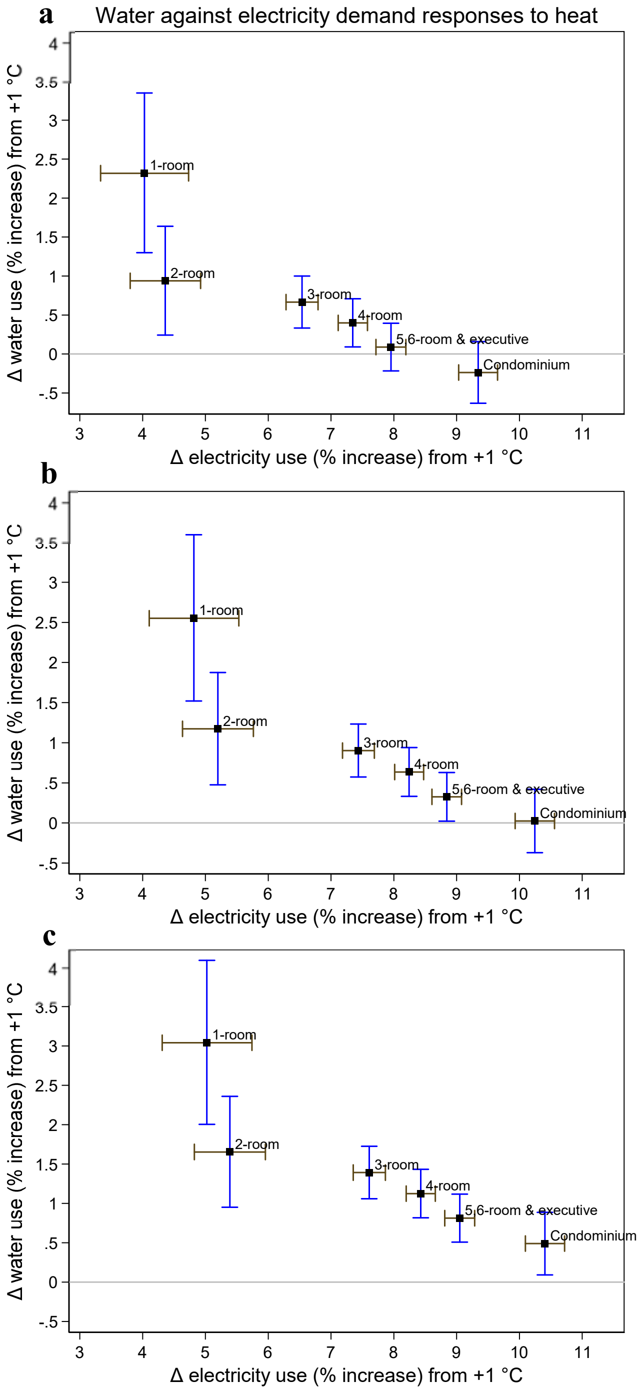

Departing from Fig. 5a's specification (in the main text) in each case, I drop controls for: **a.,** Average relative humidity and average dew point depression, **b.,** Average wind speed, and **c.,** Average PM2.5. Each panel reports 95% confidence intervals for the percent increase in water demand against the percent increase in electricity demand, by apartment type, for a +1 °C variation.

Supplementary Fig. 7. Robustness to variation in controls

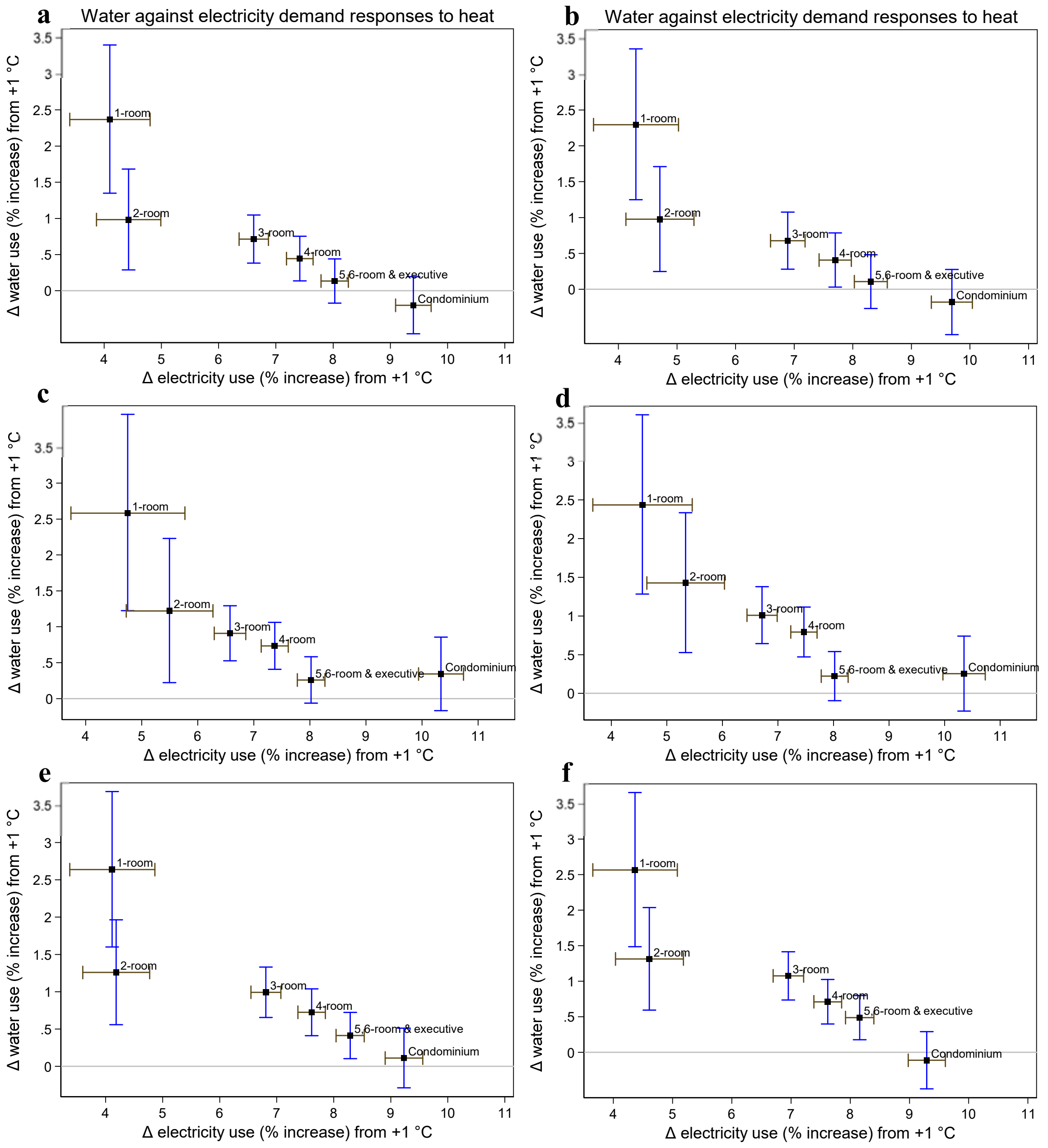

I take Fig. 5a's specification (in the main text) as the point of departure. **a.,** Drop average dew point depression (and keep average relative humidity). **b.,** Environmental controls enter flexibly via bins rather than linearly, with bin width: (i) 2% for average relative humidity, as in 72-74%; (ii) 1 °C for average dew point depression, as in 3-4 °C; (iii) 1 km h^{-1} for average wind speed, as in 6-7 km h^{-1}; (iv) 2 mm day^{-1} for average precipitation, as in 2-4 mm day^{-1}; and (v) 10 μg m^{-3} for average PM2.5, as in 15-25 μg m^{-3}. **c., d.,** Include interactions between apartment-type indicators and the economy's overall unit labor cost or average wage. **e.,** Include interactions between apartment-type indicators and the electricity price in the electricity equation (the water

price was constant in 2012-15). **f.**, Include interactions between apartment-type indicators and PM2.5, allowing for households' avoidance behavior to PM2.5 to vary by apartment type. Each panel reports 95% confidence intervals for the percent increase in water demand against the percent increase in electricity demand, by apartment type, for a +1 °C variation.

Supplementary Fig. 8. Robustness to allowing for endogenous PM2.5 and temperature.

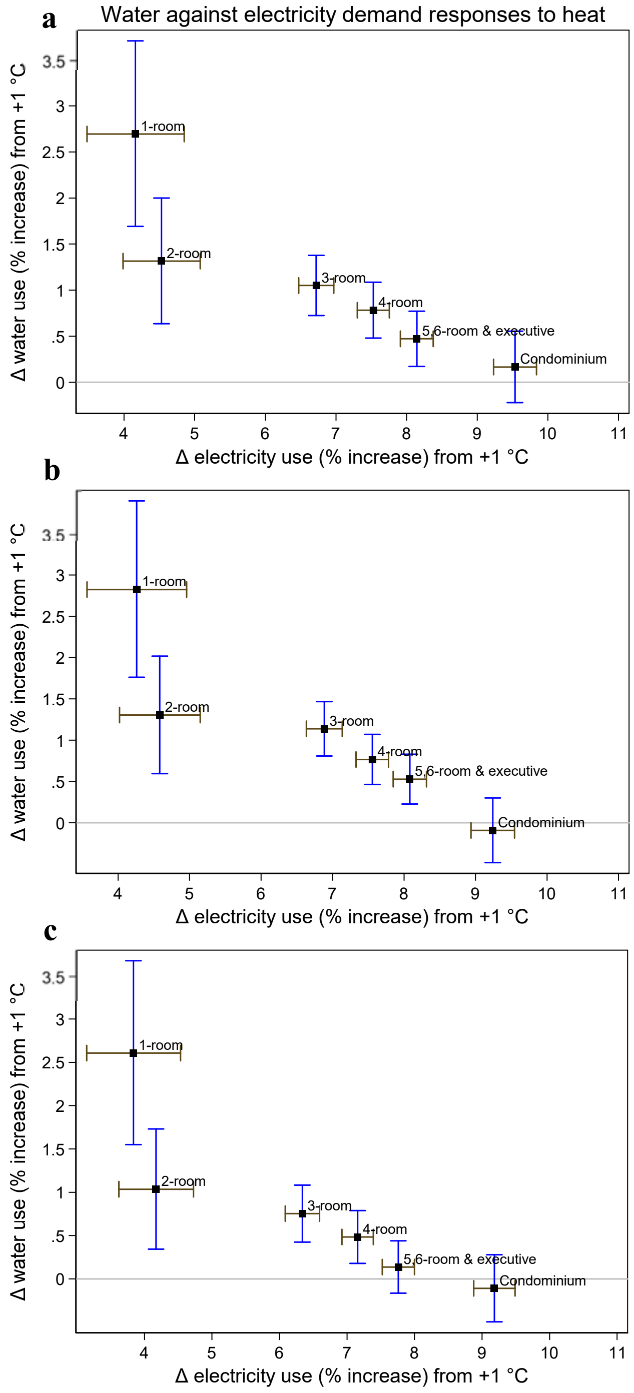

Taking Fig. 5a's specification (in the main text) as the point of departure: **a.,** PM2.5 is instrumented with Southeast Asia fire activity variables and Singapore thermal gradients and wind direction (as in specification 6 of Table 1 in the main text). **b.,** Similar specification as in **a** now including interactions between apartment-type indicators and PM2.5 (similarly, PM2.5 instruments), thus allowing for households' avoidance behavior to PM2.5 to vary by apartment type. **c.,** Similar specification as in **a** now instrumenting for temperature (as measured by MSS) with temperature

measured by an independent source (NUSG; I specify interactions between each temperature variable and apartment-type indicators). Each panel reports 95% confidence intervals for the percent increase in water demand against the percent increase in electricity demand, by apartment type, from a +1 °C variation.

Supplementary Fig. 9. Robustness to average temperature entering nonlinearly via a quadratic term

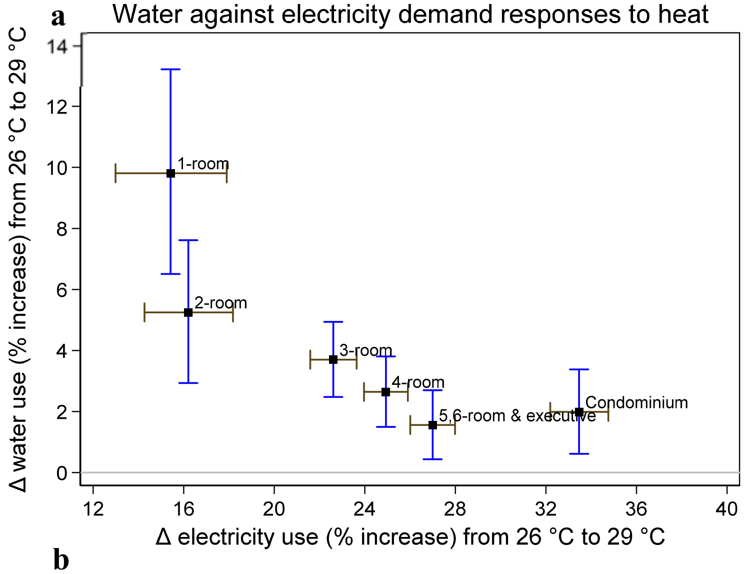

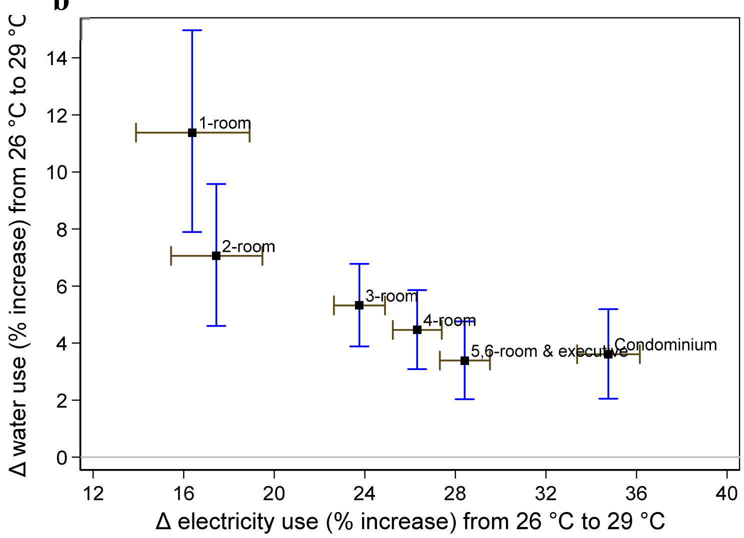

Departing from Fig. 5a's specification (in the main text), I progressively include: **a.,** Interactions between apartment-type indicators and the square of average temperature, and, additionally, **b.,** The square of each environmental control (average relative humidity, average dew point depression, average wind speed, average precipitation, and average PM2.5). The empirical support for average-period temperature is 26.1 to 29.1 °C, so I report 95% confidence intervals for the percent increase in water demand against the percent increase in electricity demand, by apartment type, for a 26.1 to 29.1 °C shift in average temperature.

Supplementary Fig. 10. Distribution of residential meter reading dates in the sample

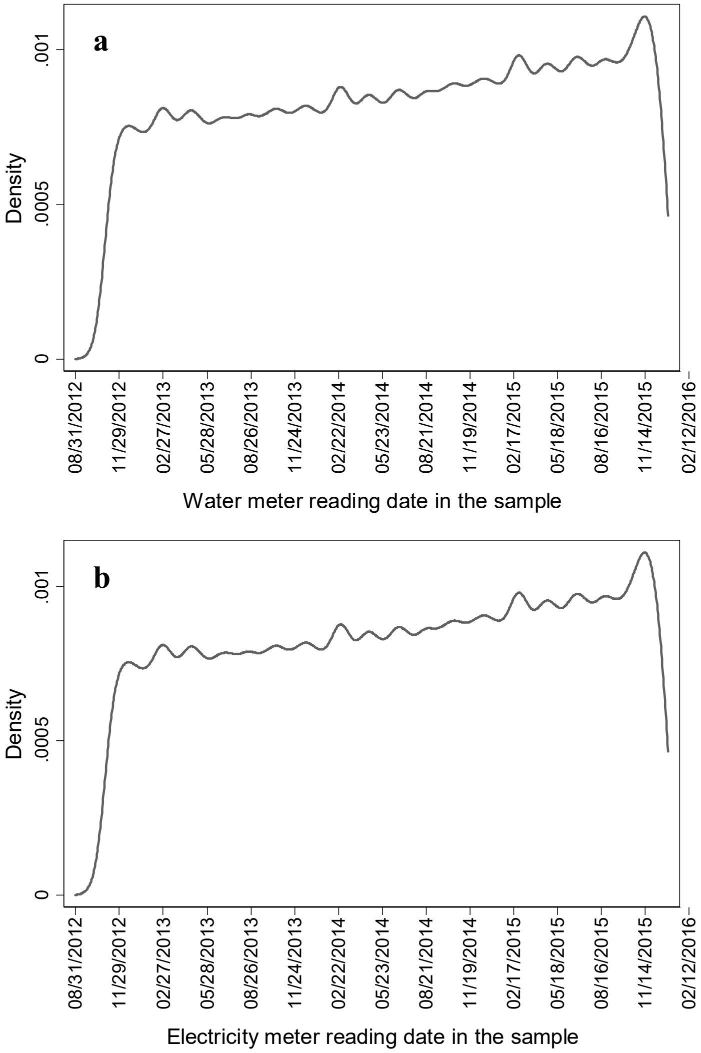

Meter reading dates for **a.,** water, and **b.,** electricity. An observation is a household by usage period among active accounts in the billing period from September 2012 to December 2015. Source: SP Services microdata.

Supplementary Fig. 11. Distribution of duration of actual use observations between successive meter readings in the sample.

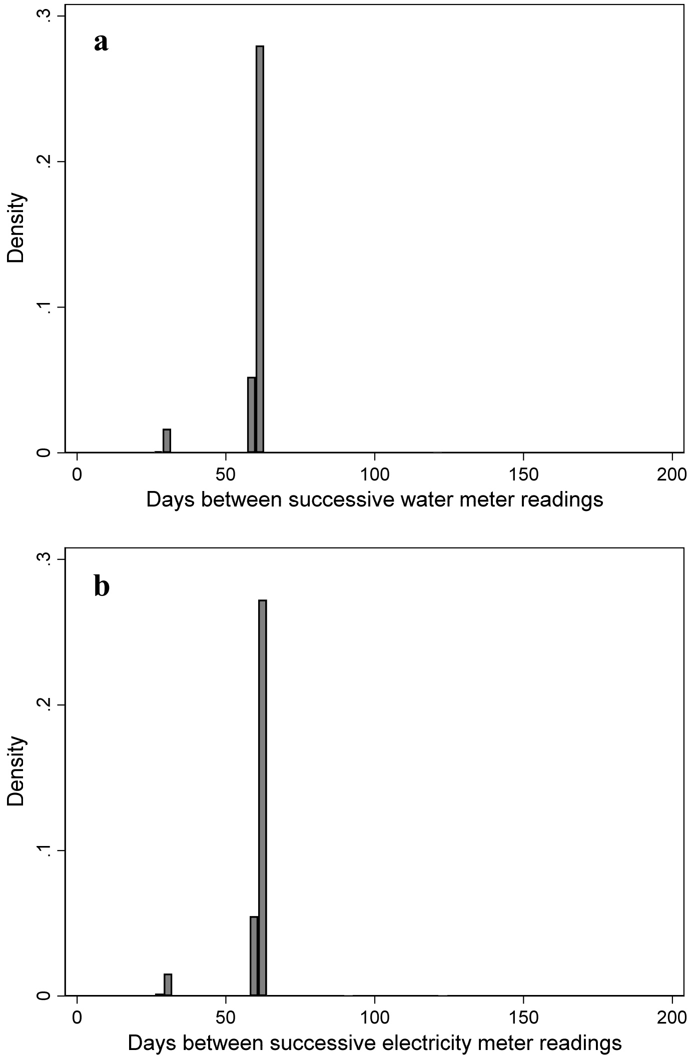

An observation is a household by usage period. The density at 59-62 days, characterized by a meter reading frequency of once every 2 months by an SP Services meter reader, is 94% for both **a.,** water use observations, and **b.,** electricity use observations. The density at 28-31 days, typically due to some customers calling in to report actual readings within one month of a meter reader's visit, is 5% for both water use observations and electricity use observations. The modal duration in each distribution is 61 days, with a 63% density for both water use observations and electricity use observations. Duration is shown up to 200 days for better visualization. Source: SP Services microdata.

Supplementary Table 1. Summary statistics for water and electricity use in the full sample and by apartment type subsample

Variable	N	Mean	Std. Dev.	p1	Median	p99
**Water use (m^3 $month^{-1}$)**						
All apartment types	2,042,026	17.1	12.3	0.3	14.8	56.9
1-room apartment	46,891	9.8	9.5	0.0	7.7	41.5
2-room apartment	65,195	12.7	10.5	0.0	10.3	47.9
3-room apartment	393,514	14.4	10.5	0.2	12.3	49.5
4-room apartment	670,596	18.0	11.5	0.6	15.9	55.3
5- or 6-room & executive apartment	528,054	19.4	12.0	0.9	17.3	58.3
Condominium apartment	337,776	16.5	15.0	0.1	13.6	71.0
**Electricity use (kWh $month^{-1}$)**						
All apartment types	2,022,845	413.2	301.5	21.1	349.4	1,434.6
1-room apartment	41,689	133.5	99.6	1.0	115.6	497.7
2-room apartment	56,479	186.5	119.7	2.0	164.3	601.5
3-room apartment	390,968	281.7	184.9	18.2	243.0	850.3
4-room apartment	667,581	379.0	205.3	34.4	340.8	1,013.7
5- or 6-room & executive apartment	529,182	467.2	248.4	47.9	422.5	1,233.4
Condominium apartment	336,946	621.6	483.1	22.6	515.4	2,379.8
**Water-to-electricity use ratio (m^3 kWh^{-1})**						
All apartment types	2,002,603	0.053	0.233	0.005	0.041	0.200
1-room apartment	41,116	0.113	0.649	0.002	0.064	0.726
2-room apartment	55,743	0.080	0.290	0.003	0.057	0.397
3-room apartment	387,634	0.061	0.179	0.006	0.048	0.236
4-room apartment	662,700	0.056	0.221	0.010	0.045	0.185
5- or 6-room & executive apartment	523,679	0.048	0.190	0.009	0.040	0.154
Condominium apartment	331,731	0.033	0.261	0.001	0.025	0.135

Notes: An observation is a household by usage period among active accounts in the billing period from September 2012 to December 2015. SP Services randomized—a 1-in-10 random sample—among all accounts that were active when the extraction code went into production, in August 2015. For each household in the random sample, SP Services provided usage by monthly billing cycle going back to September 2012, and subsequently tracked these accounts to December 2015. A string variable in the microdata informs apartment type, taking on the “values”: HDB01 = 1-room apartment, HDB02 = 2-room apartment, HDB03 = 3-room apartment, HDB04 = 4-room apartment, HDB05 = 5-room apartment, HDB06 = 6-room apartment, HDBEX = executive apartment, PTEAP = condominium apartment. HDB06 apartments are rare (only 51 identifiers in the microdata). The labels “HDB” and “PTE” denote the lead contractor for the development of apartments that are sold to individuals, whether the Housing Development Board or a private condominium developer. Source: SP Services microdata.

Supplementary Table 2. Household characteristics by apartment type

Mean annual household income per person	1000 US$			
Living in 1- or 2-room apartments	9.3			
Living in 3-room apartments	20.9			
Living in 4-room apartments	23.9			
Living in 5-room and executive apartments	29.7			
Living in condominium apartments	68.9			
**Mean household size (occupancy)**	**Persons**			
Living in 1- or 2-room apartments	2.1			
Living in 3-room apartments	2.7			
Living in 4-room apartments	3.6			
Living in 5-room and executive apartments	3.9			
Living in condominium apartments	3.4			
**Mean utility consumption per person**	**Water (L day^{-1} $person^{-1}$)**	**Electricity (kWh day^{-1} $person^{-1}$)**		
Living in 1- or 2-room apartments	182.3	2.6		
Living in 3-room apartments	177.8	3.5		
Living in 4-room apartments	166.7	3.5		
Living in 5-room and executive apartments	165.8	4.0		
Living in condominium apartments	161.8	6.1		
**Household penetration of durable goods (%)**	**Refrigerator**	**Washing machine**	**Air conditioner**	**Car**
Living in 1- or 2-room apartments	93.0	73.9	13.5	2.4
Living in 3-room apartments	98.4	93.6	57.8	14.7
Living in 4-room apartments	98.7	97.4	75.9	33.1
Living in 5-room and executive apartments	98.9	98.2	87.9	55.0
Living in condominium apartments	99.8	99.2	98.8	78.5

Notes: The unit of study is a resident household in Singapore. Mean annual household income per person (1000 US$ at 1.26 SG$ per 1 US$) includes income from all sources. I calculate utility consumption per person, by apartment type, as the mean use across household by period observations in the utility usage microdata divided by the mean household size (reported in the 2012/13 HES). Unlike the utility usage microdata, the 2012/13 HES does not report separately on 1-room apartments and 2-room apartments, only jointly. I convert utility consumption per person into a daily rate. Sources: 2012/13 Household Expenditure Survey, SP Services microdata. Source data (except SP Services microdata) are provided as a Source Data file.

Supplementary Table 3. Summary statistics for environmental variables

Variable	N	Mean	Std. Dev.	Min	Max
**Weather in Singapore, *W***					
Temperature at the surface, source MSS, daily mean (°C)	1,248	27.7	1.1	23.7	30.5
Temperature at the surface, daily maximum (°C)	1,248	31.9	1.5	25.0	34.6
Temperature at the surface, daily minimum (°C)	1,248	24.8	1.1	21.4	27.8
Temp. at surface, independent source NUSG, daily mean (°C)	1,248	27.4	1.1	23.4	30.3
Precipitation, daily total > 0 mm (yes=1)	1,248	0.7	0.5	0	1
Precipitation, daily total (mm)	1,248	6.4	10.0	0	73.9
Wind speed at the surface, daily mean (km h^{-1})	1,248	7.2	2.0	3.3	18.6
Wind speed at the surface, daily maximum (km h^{-1})	1,248	30.8	5.3	13.1	53.7
Relative humidity, daily mean (%)	1,248	75.6	5.7	54.7	96.1
Dew point depression at the surface, 8 am (°C)	1,245	3.8	2.3	0	36
**Air pollution (particles) in Singapore, *PM***					
PM2.5, North, 24-hour mean (µg m^{-3})	1,225	22.2	20.1	5.5	286
PM2.5, East, 24-hour mean (µg m^{-3})	1,225	20.9	20.2	5.8	282
PM2.5, South, 24-hour mean (µg m^{-3})	1,225	20.9	22.5	4.8	304
PM2.5, West, 24-hour mean (µg m^{-3})	1,225	22.0	21.4	4.0	256
PM2.5, Center, 24-hour mean (µg m^{-3})	1,225	19.2	17.9	4.6	247
**Atmospheric ventilation/stagnation in Singapore, *A***					
Atm. temp. gradient surface to 1000 mbar, 8 am (°C/100 m)	1,244	-0.2	0.9	-4.62	4.32
Atm. temp. gradient, surface to 925 mbar, 8 am (°C/100 m)	1,239	-0.6	0.2	-1.01	0.21
Atm. temp. gradient, 925 to 850 mbar, 8 am (°C/100 m)	1,237	-0.5	0.1	-0.87	-0.03
Wind direction at the surface, from north, 8 am (yes=1)	1,245	0.4	0.4	0	1
Wind direction at the surface, from east, 8 am (yes=1)	1,245	0.3	0.3	0	1
Wind direction at the surface, from south, 8 am (yes=1)	1,245	0.2	0.40	0	1
Wind direction at the surface, from west, 8 am (yes=1)	1,245	0.2	0.33	0	1
**Spatially aggregated fire activity in Southeast Asia, *F***					
Inverse-distance-weighted radiative power (MW km^{-1})	1,248	26.6	52.5	0.1	929.5
Direction-difference-weight. radiative power (MW×1000)	1,245	7.4	11.7	0.0	130.8
Inv.-dist. × direction-diff. weigh. radiative power (MW km^{-1})	1,245	10.3	25.5	0.0	435.2

Notes: An observation is a day in the period August 1, 2012 to December 31, 2015. PM2.5 concentrations are available from August 24, 2012 to March 31, 2014, and derived from the Pollutant Standards Index thereafter. Sources: Temperature, precipitation, and wind speed: Meteorological Service Singapore, averaging across Admiralty, Khatib, Changi, Sentosa, Tengah, and Ang Mo Kio sites. Relative humidity and temperature: NUS Geography Weather Station, NUSG, Kent Ridge site. PM2.5: National Environment Agency, available for five districts of Singapore. Dew point depression, atmospheric temperature gradients, and wind direction: NOAA Integrated Global Radiosonde Archive, IGRA Version 2, at reference latitude 1.3667 and longitude 103.9833. Fires: NASA Fire Information for Resource Management System, FIRMS. I spatially aggregate over individual hotspots detected -16 to +16 latitude degrees and -16 to +16 longitude degrees from Singapore's geographic centroid, at latitude 1.3689 and longitude 103.8013. Three weights capture proximity to Singapore. First, the inverse distance from the hotspot's location to Singapore's centroid. Second, the cosine of the difference between wind direction in Singapore and the initial bearing from Singapore's centroid to the hotspot's location, where this wind

direction-initial bearing difference is not to exceed 90 degrees in absolute value (the cosine weight is bounded from below by zero). Third, the interaction between the first two weights. Source data are provided as a Source Data file.

Supplementary Table 4. Alternative heat covariate in the household demand model

Point estimate (and standard error in parentheses) are expressed in log points.	(1) only HH FE, OLS	(2) w/ time FE, OLS	(3) w/ weather, OLS	(4) w/ PM2.5, OLS	(5) trend, not year, OLS	(6) PM2.5 endog., 2SLS
**A. Dependent var.: Log water use** (m^3 $month^{-1}$)						
Prop. days w/ mean temperature≥28.5 °C (%)	0.0336***	0.0358***	0.0325***	0.0222***	0.0148***	0.0236***
	(0.0015)	(0.0031)	(0.0034)	(0.0035)	(0.0035)	(0.0034)
Number of observations	2,001,389	2,001,389	1,998,127	1,998,127	1,998,127	1,998,127
Number of regressors (not counting HH FE)	1	23	27	28	26	28
Number of households	121,480	121,480	121,480	121,480	121,480	121,480
R^2 [First-stage F-statistic, excluded instr.]	0.830	0.830	0.830	0.830	0.830	[946,413]
Mean value of usage in sample	16.66	16.66	16.66	16.66	16.66	16.66
**B. Depend. var.: Log electricity use** (kWh $mo.^{-1}$)						
Prop. days w/ mean temperature≥28.5 °C (%)	0.3105***	0.2223***	0.1844***	0.1633***	0.1586***	0.1594***
	(0.0013)	(0.0022)	(0.0024)	(0.0025)	(0.0025)	(0.0024)
Number of observations	1,982,472	1,982,472	1,979,238	1,979,238	1,979,238	1,979,238
Number of regressors (not counting HH FE)	1	23	27	28	26	28
Number of households	120,099	120,099	120,099	120,099	120,099	120,099
R^2 [First-stage F-statistic, excluded instr.]	0.884	0.885	0.885	0.885	0.885	[947,822]
Mean value of usage in sample	405.91	405.91	405.86	405.86	405.86	405.86
**C. Dependent var.: Log water use** (m^3 $month^{-1}$)						
Prop. days w/ mean temperature≥29 °C (%)	0.0402***	0.0180***	0.0139***	0.0195***	0.0221***	0.0190***
	(0.0021)	(0.0037)	(0.0037)	(0.0037)	(0.0039)	(0.0036)
Other diagnostics omitted (similar to panel A)						
**D. Depend. var: Log electricity use** (kWh $mo.^{-1}$)						
Prop. days w/ mean temperature≥29 °C (%)	0.4194***	0.1659***	0.1396***	0.1543***	0.1527***	0.1574***
	(0.0018)	(0.0027)	(0.0026)	(0.0027)	(0.0028)	(0.0026)
Other diagnostics omitted (similar to panel B)						

Notes: The table reports estimates for 24 water or electricity use regressions. An observation is a household by usage period in the 2012-2015 utility usage microdata. The dependent variable is log water use (m^3 $month^{-1}$) in panels **A** and **C**, and log electricity use (kWh $month^{-1}$) in panels **B** and **D**. The key regressor is the proportion of days with daily mean temperature above 28.5 °C in panels **A** and **B** (empirical support 0 to 83%), or above 29 °C in panels **C** and **D** (empirical support 0 to 77%). Proportions are taken over the same days that are concurrent to each usage observation. Other notes to Table 1 in the main text apply here exactly. OLS regressions in columns **1** to **5**, 2SLS regressions in column **6**. Standard errors (se), in parentheses, clustered by household. ***, **, * denote significance at the 0.01, 0.05 and 0.1 levels, respectively.

Supplementary Table 5. Tests of equality of heat-water responses and heat-electricity responses for the different pairs of apartment types

**A. Pairwise equality test for the temperature effect on water use**					
**Test statistic** (log points) **[p-value]**	2-room apartments	3-room apartments	4-room apartments	5,6-room & executive	Condominium apartments
1-room apartments	0.0135** [0.0178]	0.0161*** [0.0008]	0.0188*** [0.0001]	0.0219*** [<0.0001]	0.0249*** [<0.0001]
2-room apartments		0.0026 [0.4282]	0.0053 [0.1038]	0.0084*** [0.0099]	0.0114*** [0.0011]
3-room apartments			0.0027*** [0.0087]	0.0058*** [<0.0001]	0.0088*** [<0.0001]
4-room apartments				0.0031*** [0.0001]	0.0061*** [<0.0001]
5,6-room and executive apartments					0.0030** [0.0432]

**B. Pairwise equality test for the temperature effect on electricity use**					
**Test statistic** (log points) **[p-value]**	2-room apartments	3-room apartments	4-room apartments	5,6-room & executive	Condominium apartments
1-room apartments	-0.0035 [0.3986]	-0.0243*** [<0.0001]	-0.0318*** [<0.0001]	-0.0375*** [<0.0001]	-0.0503*** [<0.0001]
2-room apartments		-0.0207*** [<0.0001]	-0.0283*** [<0.0001]	-0.0340*** [<0.0001]	-0.0467*** [<0.0001]
3-room apartments			-0.0075*** [<0.0001]	-0.0132*** [<0.0001]	-0.0260*** [<0.0001]
4-room apartments				-0.0057*** [<0.0001]	-0.01845*** [<0.0001]
5,6-room and executive apartments					-0.0128*** [<0.0001]

Notes: The table reports the p-value, in square brackets, of pairwise equality tests for the apartment-type specific temperature coefficients for **A.,** the regression of log water use, and **B.,** the regression of log electricity use, reported in Fig. 5a in the main text. The test statistic, in log points, is the temperature coefficient for the apartment type shown along the rows minus the temperature coefficient for the apartment type shown across the columns. ***, **, * denote significance at the 0.01, 0.05 and 0.1 levels, respectively.

Supplementary Table 6. Heat-water response is lower in markets with higher average electricity use

Market-level electricity by temperature interaction. Point estimates and standard errors are expressed in log points in (1) to (3) and in m^3 $month^{-1}$ in (4).	(1) Market-level Electricity	(2) Market-level Log Electricity	(3) Market-level Electricity per person	(4) Market-level Electricity
**Dependent variable:**	**Log water use**	**Log water use**	**Log water use**	**Water use**
Mean temperature over usage period (°C)	0.0150***	0.0596***	0.0149***	0.1662***
	(0.0025)	(0.0111)	(0.0027)	(0.0226)
Mean temperature (°C) × market-level electricity use (10^3 kWh $mo.^{-1}$ HH^{-1})	-0.0201***			-0.2341***
	(0.0045)			(0.0428)
Mean temperature (°C) × market-level **log** electricity use (kWh $mo.^{-1}$ HH^{-1})		-0.0089***		
		(0.0018)		
Mean temperature (°C) × market-level electricity use **per person** (10^3 kWh $mo.^{-1}$ $person^{-1}$)			-0.0686***	
			(0.0185)	
Number of observations (121,480 HHs)	1,998,130	1,998,130	1,998,130	1,998,645
Mean value of usage in sample (m^3 $month^{-1}$)	16.66	16.66	16.66	16.66

Notes: The table reports estimates for four water use regressions. An observation is a household (HH) by usage period in the 2012-2015 utility usage microdata. The dependent variable is log water use (or water use, m^3 $month^{-1}$, in specification 4). The regressors of interest are average daily mean temperature (°C) and its interaction with average electricity use for the household's geographic area and apartment type, computed as mean electricity use over all household and time varying observations within postal code by apartment type in the sample. This time-invariant variable has empirical support 6 to 1,104 kWh $month^{-1}$ and shifts by apartment type (six) within postal code (73 in total). I divide the interaction variable by 1000 in order to scale up its coefficient by this factor. In specification 3, average electricity use is adjusted for the mean household size within apartment type. As in Table 1 in the main text, all regressions include controls for household, time (month, year, day-of-the-week, public holidays, school holidays), weather (relative humidity, dew point depression, wind speed, precipitation) and PM2.5. Average market-level electricity use is included but subsumed in the household fixed effects. OLS regressions. Standard errors, in parentheses, clustered by household. ***, **, * denote significance at the 0.01, 0.05 and 0.1 levels, respectively.

Supplementary Table 7. Heat-relief behaviors stated by Singapore households

A

It is a **very hot weekday in Singapore this September**. Your **daily routine has been normal**, attending school or work, running errands, going to the community centre, and so on. **Which of the following may apply to your home that day or evening? Please tick at most three (one, two or three) statements that are more likely to apply:**	Number of respondents (of N=311 in total)	%
Due to the heat, at home we/I **shut the windows and turned on the air conditioner**, compared to a cooler day.	113	36%
Due to the heat, at home we/I **turned on the electric fan**, compared to a cooler day.	187	60%
Due to the heat, at home we/I **showered more often or for longer**, compared to a cooler day.	121	39%
Due to the heat, at home I **washed my face more often or for longer**, compared to a cooler day.	55	18%
Due to the heat, at home **I put wet towels in the refrigerator to place on my body once cool**, compared to a cooler day.	15	5%
Due to the heat, **I spent more time at home**, compared to a cooler day.	135	43%
Due to the heat, at home we/I **put more dirty/sweaty clothes in the washing machine**, compared to a cooler day.	57	18%
Other (open-ended response) ________________________________	1‡	0%
None of the statements above are more likely to apply at home due to the heat, compared to a cooler day.	7	2%

‡ One respondent wrote: "We drink more water each day"

B

Age (years)	20-24<br>8%	25-34<br>22%	35-44<br>24%	45-54<br>21%	55-64<br>13%	65+<br>12%
Position in the household (HH) (select **one**)	Head<br>55%	Spouse<br>17%	Son/daughter<br>22%	Parent of HH head<br>2%	Flat-mate/ room-mate<br>4%	
Type of dwelling / premises I live in (select **one**)	1,2-room apartment<br>5%	3-room apartment<br>26%	4-room apartment<br>23%	5,6-room/ exec apt.<br>23%	Condominium apartment<br>22%	
Appliances at home (**tick all that apply**)	Air conditioner<br>85%	Electric fan<br>96%	Shower<br>97%	Washing machine<br>95%	Bath tub<br>20%	

Notes: I implemented the survey on the Qualtrics online platform starting on September 11, 2018 (date of the "soft launch") and ending 12 days later. After an attention check, respondents were first primed with a picture of a person sweating under the sun. Next, respondents were asked to consider "a very hot weekday in Singapore this September… (in which their) daily routine has been normal…" Respondents were then asked to choose at most three heat-relief strategies that would more likely apply to their home. The survey also highlighted the words highlighted in the panels. **A.,** Number and proportion of respondents selecting the different alternatives. These alternatives appeared in random order by respondent, except for "other" and "none," which appeared at the end of the list of alternatives. **B.,** Distribution of demographic characteristics over respondents. These characteristics were surveyed in a final question, in the order reported. The list of home appliances appeared in random order by respondent. Source data are provided as a Source Data file.
